# How does genetic risk information for Lynch syndrome translate to risk management behaviours?

**DOI:** 10.1186/s13053-016-0061-6

**Published:** 2017-01-05

**Authors:** Emma Steel, Andrew Robbins, Mark Jenkins, Louisa Flander, Clara Gaff, Louise Keogh

**Affiliations:** 1 Centre for Health Equity, The University of Melbourne, Melbourne, Australia; 2 Centre for Epidemiology and Biostatistics, The University of Melbourne, Melbourne, Australia; 3 Walter and Eliza Hall Institute, Melbourne, Australia

**Keywords:** Lynch syndrome, Predictive genetic testing, Patient-clinician communication, Screening colonoscopy

## Abstract

**Background:**

There is limited research on why some individuals who have undergone predictive genetic testing for Lynch syndrome do not adhere to screening recommendations. This study aimed to explore qualitatively how Lynch syndrome non-carriers and carriers translate genetic risk information and advice to decisions about risk managment behaviours in the Australian healthcare system.

**Methods:**

Participants of the Australasian Colorectal Cancer Family Registry who had undergone predictive genetic testing for Lynch syndrome were interviewed on their risk management behaviours. Transcripts were analysed thematically using a comparative coding analysis.

**Results:**

Thirty-three people were interviewed. Of the non-carriers (*n* = 16), 2 reported having apparently unnecessary colonoscopies, and 6 were unsure about what population-based colorectal cancer screening entails. Of the carriers (*n* = 17), 2 reported they had not had regular colonoscopies, and spoke about their discomfort with the screening process and a lack of faith in the procedure’s ability to reduce their risk of developing colorectal cancer. Of the female carriers (*n* = 9), 2 could not recall being informed about the associated risk of gynaecological cancers.

**Conclusion:**

Non-carriers and female carriers of Lynch syndrome could benefit from further clarity and advice about appropriate risk management options. For those carriers who did not adhere to colonoscopy screening, a lack of faith in both genetic test results and screening were evident. It is essential that consistent advice is offered to both carriers and non-carriers of Lynch syndrome.

## Background

There are various genetic syndromes related to colorectal cancer (CRC). One of the most common is Lynch syndrome (LS), which accounts for 1 – 3% of all CRCs [1]. LS develops as a result of an autosomal dominant germline mutation in one of a group of DNA mismatch repair (MMR) genes, notably the following four: MLH1, MSH2, MSH6 and PMS2. When a MMR gene mutation is identified in an individual with CRC (i.e. a carrier), predictive genetic testing is then available to first degree relatives. Without preventive measures, the development of CRC amongst LS affected individuals is estimated at a lifetime risk of 10 – 74%, depending on sex and the MMR gene mutated [2, 3]. Female carriers are also at an increased risk of gynaecological cancers (GC), with an estimated lifetime risk for endometrial cancer of 14 – 71%, and 4 – 20% for ovarian cancer [2, 3]. Individuals with a pathogenic mutation for LS also have an increased risk of gastric cancers (3 – 13%) and other extra-colonic malignancies such as brain tumours and skin cancer [2–5] . Routine screening in the form of annual or biennial colonoscopy from age 25 (or 5 years earlier than the youngest CRC diagnosis in the family) is the standard recommendation for individuals with LS in Australia [6, 7]. Regular colonoscopy screening has been shown to reduce the risk of developing CRC [8] and delay the age of onset by more than 10 years [9], with CRC mortality rates reduced by up to 65% among LS individuals [7]. Colorectal surgical intervention is generally not recommended to individuals with LS until an index CRC develops. Screening recommendations are less clearly established for associated GC, with a suggestion that women should consider starting annual pelvic ultrasounds with endometrial sampling between 30 and 35 years of age for the detection of endometrial cancer, however the evidence of benefit is weak [10]. To date, no benefit has been shown for ovarian cancer screening [11]. The only effective intervention to reduce the risk of GC in women is prophylactic hysterectomy and bilateral salphingo-oophorectomy (BSO) from age 40 years, or once they have completed childbearing [12, 13].

An individual who tests negatively for a known family MMR gene mutation (i.e. a non-carrier) is considered to be at population risk for all LS-associated cancers, and is therefore advised to follow population-based CRC screening recommendations [14]. Recommended population-based CRC screening in Australia is a non-invasive immunochemical faecal occult blood test (FOBT) every 1 – 2 years, commencing at age 50 [15]. As part of the National Bowel Cancer Screening Program, a free FOBT kit is currently sent in the mail to all Australians turning 50, 55, 60, 65, 70 and 74 [16]. Given the risk of adverse event such as perforation [17, 18], and associated cost of the procedure, screening colonoscopies are not recommended to those at population risk of CRC [19], and are generally only used to investigate a positive FOBT result (i.e. blood is detected).

Previous research has linked personal genetic mutation results with the adoption of more appropriate screening intervals, such that carriers increase screening frequency and non-carriers decrease screening intervals if screening previously at a rate higher than recommended [20–26]. One of the first studies to evaluate long-term follow-up for individuals who have been tested for LS demonstrated through 3-year longitudinal data that the majority of those who received genetic risk information screened appropriately according to their carrier status [27]. Of 19 carriers in the study, all had a colonoscopy within 3 years, the majority of whom screened in the first 12 months. Of 54 non-carriers, only 7% had a colonoscopy within the 2 years before the 3-year follow-up. Nine of 13 female carriers (69%) reported having had a pelvic ultrasound in the previous 2 years, including endometrial sampling in seven cases. As noted in many previous studies, psychological distress returned to baseline levels within 12 months of receiving results and was maintained for 2 years in carriers. For non-carriers, levels of psychological distress steadily decreased over 3 years. A recent study by Esplen et al. [28] also found that in the long term individuals tend to adapt to their genetic test result, with self-reported screening being much higher in carriers than non-carriers. Studies to date have been unable to explain why some non-carriers continue to have unnecessary invasive screening when they are considered to be at population risk of CRC, nor do they report on women’s uptake of measures to address their increased risk of GC.

Given how little is known about why some individuals who have undergone predictive genetic testing for LS do not adhere to screening recommendations, this study employed a qualitative methodology with a population-based sample to explore how LS carrier status translates to risk management behaviour. Specific research interests included why some non-carriers continue to have unnecessary invasive screening, and why some carriers do not follow screening recommendations.

## Methods

### Recruitment

The Australasian Colorectal Cancer Family Registry (ACCFR) studies a large number of population-based Victorian residents recently diagnosed with CRC (within 2 years) and attendees to family cancer clinics throughout Australia and New Zealand, as well as their relatives, referred because of suspicion of having a genetic syndrome [29]. CRC tumours were obtained and tested for mismatch repair deficiency. In a course of research setting, ACCFR participants with a mismatch deficient tumour were then tested for germline mutations in the mismatch repair genes. ACCFR participants and their family members were offered the chance to have predictive genetic testing through their nearest Family Cancer Clinic (FCC).

For this study, individuals were identified through the ACCFR database according to the following criteria:

- Member of a family identified as carrying a mutation in one of the following MMR genes: MLH1, MSH2, MSH6 or PMS2
- Underwent genetic testing and received results for LS either independently or after being offered results by the ACCFR, no more than 10 years ago
- No personal history of CRC
- Aged 18 – 70 years
- Australian resident

Ethics approval was obtained through The University of Melbourne Human Ethics Sub-Committee. Based on the above criteria, over 300 ACCFR participants were eligible to be approached about the study. Forty-three individuals from separate families were selected based on their MMR mutation status, with equal numbers of positive and negative genetic test results sampled. Each eligible individual was sent a participant information and consent form and followed up by phone to discuss whether or not they wished to participate. Interviews were arranged at a time and place convenient to the participant. Written informed consent was obtained on the day of the interview. Data collection continued until saturation of the main themes was determined.

### Data Collection

Thirty-three interviews were conducted, predominantly in participant’s homes in both rural and metropolitan regions in the states of Victoria and Queensland. Data collection consisted of an audio-recorded, semi-structured interview on the topic of genetic testing for LS. Interviews lasted between 60 and 90 min. General themes included: cancer experience, genetic counselling experience, communication around genetic test result, and screening behaviours following genetic testing. The interviews were transcribed verbatim and all identifying details were removed from the transcripts.

### Data analysis

NVivo 10 software was utilised to manage and code the interview data [30]. Transcripts were read multiple times and coded into the themes identified. A comparative method of coding and analysis was conducted, and disparities were discussed until consensus was met. Non-carriers were analysed separately from the carriers in order to determine how each group utilised the knowledge of their genetic status to inform their risk management practices, and how they found the process of organising their screening. Additionally, female carriers were analysed in relation to their knowledge and decision-making regarding risk management for GC.

## Results

Of the 33 participants interviewed, 16 (48%) had tested negatively (non-carriers) and 17 (52%) had tested positively (carriers) for a mutation in one of the MMR genes. General characteristics of the sample are reported in Table 1. All participant names presented in this paper are pseudonyms.

**Table 1:** Participant characteristics

Characteristics	Number (%) *n* = 33
Sex	
Male	15 (45)
Female	18 (55)
Age at interview (years)	48 (mean)
Years since testing	5.4 (mean), 2 weeks – 10 years (range)
Mutation status	
Positive (carrier)	17 (51)
Negative (non-carrier)	16 (49)
Relatives diagnosed with CRC	2 (mean)

### Non-carriers – CRC risk management

Seven of the 16 non-carriers (44%) reported continuing with colonoscopies since receiving a negative genetic test result, either biennially or sporadically (Tables 2 and 3). Five of these non-carriers (31%) were having surveillance colonoscopies due to a personal history of polyps (quotes 1 and 2). As illustrated by Patrick, most of these non-carriers with a personal history of polyps found reassurance in their continued colonoscopies. They understood that although their genetic status placed them at population risk of CRC, their history of polyps required them to have ongoing surveillance. However, for one non-carrier (Ella), the ongoing detection of polyps negated the potential reassurance of a negative genetic test result.

**Table 2:** Non-carriers – reported CRC risk management recommendations and what participants do in practice (*n* = 16)

Clinical recommendation	In practice
Can’t recall (8)	Population screening (2)
	No current CRC screening (6)
Population screening (1)	No current CRC screening (1)
Biennial colonoscopy (5)	Biennial colonoscopy (2)
	Biennial colonoscopy and population screening (1)
	Sporadic colonoscopies and population screening (2)
Conflicting (2)	Biennial colonoscopy (1)
	Sporadic colonoscopies and population screening (1)

**Table 3:** Quotes from non-carriers – CRC risk management

Quote no.	Participant	Quote
1.	Patrick (48, tested 2.5 years ago)	*I’ve always had polyps, I normally have a colonoscopy every two years now…Obviously it’s hereditary, but because I’ve had the gene test done, well I’m no different to the normal person in the street I suppose.*
2.	Ella (50, tested 6.5 years ago)	*I have to have colonoscopies regardless because I have continued sort of polyps, so I’ve always believed that they’re gonna find another gene so I have never completely let myself off the hook.*
3.	Jean (63, tested 6.5 years ago)	*The Doctor (Gastroenterologist) that I was working for at the time suggested I have it every two years…I think they (FCC) said five to ten years actually…He said well that’s up to you, but I would like to see you do it every two years…It’s awful, but I’d much rather do all of this to make sure I’m screened and there’s nothing there…I haven’t got the gene but…I’m not scientifically trained enough to know what the odds are or anything like that.*
4.	Greg (65, tested 6 years ago)	*I’ve got Barrett’s syndrome…When I was found not to have the gene [for Lynch syndrome] they said you can have it every two years now the colonoscopy, but I’ve got to have the gastroscopy every two years for sure…Because of the family history and the Barrett’s syndrome and all of that sort of stuff…I have them at the same time.*
5.	Michael (41, tested 2 weeks ago)	*A doctor [at the FCC] came in and was explaining that their best practice understanding is you do a faecal occult blood test every two years once you hit fifty, which I’m happy to do.*
6.	Paula (32, tested 3 years ago)	*Because my risk is still there but just the same as the normal person, then the normal screenings…I can’t really remember in terms of colonoscopies and stuff, you know starting age…That’s probably something that I can actually do a bit of homework on and find out roughly when I should be having a bit of a think about it.*
7.	Andrew (38, tested 4 years ago)	*I remember them saying that even though I don’t have the gene, I’m still in the general population’s risk level…Whether I wasn’t listening enough or haven’t read enough of the information they gave me but I guess I’m not entirely sure as to what that means in terms of the recommendation would be for my own future. I guess I’m like the average person, how often, what sort of check-ups should I have? Should I have one of the FOB whatever they call its?*
8.	Alan (41, tested 4 years ago)	*Oh I guess someone who goes there and finds out a positive result probably needs a little bit more of discussion about the consequences and what that might mean in their life. Someone who gets a negative one, I just remember it being pretty short and sharp and congratulations you’re back in the normal population, and you know, walk out.*
9.	Kay (44, tested 5 years ago)	*I get the feeling that they didn’t say much because otherwise I would have been probably, if they had have suggested still being screened I probably would have been doing that so I think that they probably didn’t say anything. They just said that we haven’t got the gene.*
10.	Meagan (33, tested 4.5 years ago)	*You’re always living as if you were going to get cancer, the inevitable. It was a bit hard to change the mindset of you’re not going to or you know you’ve got the same chance as the general population…Just to go, okay, it’s not inevitable.*
11.	Michael (41, tested 2 weeks ago)	*I got it in my head that I was going to die at thirty-six and not see my son grow up…What also dawned upon me was that suddenly I’m now part of the normal population, that bowel cancer is not something that I carry with me anymore, and I’m still getting used to that idea…You almost become protective of bowel cancer, it’s like it’s your thing…Now it’s no longer that, and it’s kind of nice.*

Only two non-carriers (13%) were having screening colonoscopies at the time of interview (quotes 3 and 4). Jean recalled conflicting recommendations, noting that the FCC suggested that she have a colonoscopy every 5-10 years, but the gastroenterologist she was working with at the time recommended biennial colonoscopies based on her family history. Greg’s reported recommendation from his gastroenterologist was also based on his family history of CRC, as well as the fact that he has Barrett’s syndrome, a known precursor to oesophageal cancer. However, the current standard screening in individuals with Barrett’s syndrome is regular gastroscopies, not colonoscopies [31]. Greg was also having FOBTs as part of population-based CRC screening.

Of the seven non-carriers who reported not having any form of CRC screening (56%), all were younger than 50 years of age at the time of interview. Only one of these non-carriers could recall that he had been recommended to have FOBTs from age 50 as per population CRC screening guidelines (quote 5). The other six non-carriers not enrolled in regular CRC screening could all recall being told that they were considered to be at population risk, but none of them knew at what age population-based screening commences or what procedure is involved (quotes 6 and 7). Some of these non-carriers recalled that their genetic results disclosure session was brief, with very little information on what they should do regarding CRC screening (quotes 8 and 9).

Two of the non-carriers who were no longer having screening colonoscopies spoke about their difficulty in accepting their non-carrier status (quotes 10 and 11). Prior to testing, both had lived for several years as though they were at high risk, perceiving that they would almost definitely develop CRC. Meagan had been tested 4.5 years previously and spoke retrospectively about her initial struggle to change her risk perception from high to population risk. Michael had received his genetic test result only 2 weeks previously and was still in the midst of accepting his population risk status. Following testing, non-carriers described having to adapt to not only a change of mindset, but also a new identity, with CRC no longer being their *“thing”*.

Overall, non-carriers expressed a range of responses to their test results. Some complied with their gastroenterologist’s recommendation for ongoing screening colonoscopies due to their family history, despite no longer requiring that level of screening. This provides evidence that for at least some gastroenterologists, a negative genetic test result does not override a family history. Those who were not having regular screening colonoscopies understood that they were at population risk, but were not aware of the recommended screening for their risk level. There was evidence that for some, adjustment to a reduced risk of CRC can take time.

### Carriers – CRC risk management

All carriers recalled being recommended to have regular screening colonoscopies. Fifteen of the 17 carriers (88%) reported having annual or 18 monthly colonoscopies (Tables 4 and 5). Three of these carriers received conflicting recommendations from the FCC and their gastroenterologist about how frequently they should be screening, and reported following the advice of their gastroenterologist (quote 12). The majority of carriers spoke about their dislike of the colonoscopy procedure, in particular the preparation involved. However, all those who regularly screened expressed faith in the relationship between screening and CRC risk reduction (quotes 13 and 14).

**Table 4:** Carriers – reported CRC risk management recommendations and what participants do in practice (*n* = 17)

Clinical recommendation	In practice
Conflicting (3)	18 monthly (2)
	Annual (1)
Annual (13)	Annual (12)
	Sporadic (1)
Biennial (1)	Sporadic (1)

**Table 5:** Quotes from carriers – CRC risk management

Quote no.	Participant	Quote
12.	Amy (32, tested 8 years ago)	*I think it was a bit conflicting, you know some say every second year up until the age of thirty I think it was, but I’ve been having annual for as long as I can remember. So yeah that seems to be a little bit grey that area – some say annually, some say every second year.*
13.	Linda (47, tested 10 years ago)	*I mean I hate having them, but you know you just go oh well, it’s locked in. I know what I need to do for that day.*
14.	Eve (52, tested 1.5 years ago)	*Never having had a polyp and then this last time there was a polyp I go, okay, that’s why I do it…Cause I need to have the polyp to create the cancer…Reassuring, that’s right. That’s why I’m doing it, to get it out*
15.	Matt (37, tested 10 years ago)	*I don’t enjoy doing it. I’ve always asked is there any other way of doing it, can I try anything else and they say, nuh, this is it…I’ve missed it a couple of times, I just don’t respond…I just feel uncomfortable doing it.*
16.	Fred (55, tested 4.5 years ago)	*They said to do it every two years, and yet I’ve taken five years. As I said to you, I’ll probably do it more now because I’m getting older and because the doctor I’m with now, I’m more comfortable with him.*
17.	Matt (37, tested 10 years ago)	*My dad keeps on drumming in my head to keep on getting tested, but I don’t know. It’s not going to solve anything…The wife, she’s cracked it a few times at me because sometimes I haven’t gone. I said if it’s gonna get me, it’s gonna get me…*[How would you describe your chance of getting bowel cancer now?] *High. Ninety percent. They (colonoscopies) can’t prevent it. It’s just getting it at an early stage, it’s not going to stop it by coming.*
18.	Fred (55, tested 4.5 years ago)	*No risk at all, because I just don’t think we know enough about it to be able to say that you’re at higher risk. Someone could have a test and say you haven’t got the gene, but then they develop another form of cancer from something else…If I had a colonoscopy tomorrow, didn’t have one for two years but then in between that two years had cancer, why didn’t it get picked up? What brings it out at what stage?*

Two carriers (12%) reported having colonoscopies sporadically. Matt discussed his dislike of the colonoscopy procedure, which put him off having them on a regular basis (quote 15). Fred reported he wasn’t having regular colonoscopies due to a poor relationship with his previous doctor (quote 16). He was asked if he would be more likely to have regular colonoscopies if they were organised on his behalf, but he didn’t think that would make much difference. However, he was hopeful to begin screening more regularly given his increased comfort with his current doctor. Despite not undertaking regular screening, Matt believed his risk of developing CRC was high and placed it at 90% (quote 17). In contrast, Fred did not perceive his risk of developing cancer to be any higher than the average person, which fits with his perception that CRC risk increases with age in LS individuals (quote 18). However, rather than focusing solely on CRC, Fred viewed cancer as a single disease of which everyone is at risk. He also expressed uncertainty about the value of the genetic test, suggesting that not enough is known about it yet to be able to place individuals at increased risk. Additionally, neither Matt nor Fred expressed faith in the procedure’s ability to reduce their risk of developing CRC.

These excerpts demonstrate that in order to adhere to screening recommendations, carriers need to believe they are at increased risk of CRC due to their LS mutation status, be able to tolerate the procedure, and have faith in screening as a means of risk reduction as well as early detection.

### Carriers – GC risk management

Female carriers (*n* = 9) were asked about their GC screening and risk management behaviours (Tables 6 and 7). Seven (78%) could recall a discussion with the FCC regarding their gynaecological cancer risk, three of whom subsequently had a prophylactic hysterectomy and BSO, and four of whom were having annual pelvic ultrasounds (quote 19). Of the four who had not had surgery, but were having annual pelvic ultrasounds, two were not aware of prophylactic hysterectomy and BSO as a GC risk management option (quote 20). Linda noted she recently attended a workshop on LS, and was questioning the effectiveness of GC screening and planned to have a discussion with her gynaecologist. Of the other two female carriers who reported having annual pelvic ultrasounds, one hoped to have children in the near future (quote 21), and one didn’t think the surgery was warranted while she was healthy (quote 22).

**Table 6:** Female carriers – reported GC risk management recommendations and what participants do in practice (*n* = 9)

Clinical recommendation	In practice
Can’t recall (2)	No current GC screening (2)
Annual pelvic ultrasound (2)	Annual pelvic ultrasound (2)
Prophylactic hysterectomy and BSO (3)	Prophylactic hysterectomy and BSO (3)
Annual pelvic ultrasound, prophylactic hysterectomy and BSO (2)	Annual pelvic ultrasound (2)

**Table 7:** Quotes from carriers – GC risk management

Quote no.	Participant	Quote
19.	Adele (41, tested 4 years ago)	*We talked about future surgeries and my options as what way to go next, it was all done straight away…She sort of said, go away and think about it…So within three months I was in hospital having a full hysterectomy…For me, prevention is better than cure.*
20.	Linda (47, tested 10 years ago)	*They (the FCC) actually gave me a contact for a Gynaecologist that dealt with people with Lynch syndrome…They said the colonoscopies would be every year and the endometrial sort of stuff would be probably once a year as well…From that Lynch workshop we had the other day, they were sort of saying that maybe that’s not the best sort of screening [for gynaecological cancers]…I’ll probably have to ask the gynaecologist to make sure that well is this the best screening, or what do they recommend?*
21.	Caroline (31, tested 8 years ago)	*[Do you think about it a lot?] More so the issue of the uterus cancer, not having kids and cause I haven’t sort of necessarily found someone, that probably gets to me a little bit more than anything.*
22.	Shelly (65, tested 1.5 years ago)	*I remember them telling me that, the specialist, that he may suggest that I have a hysterectomy…So I went and sure as eggs, that’s what he suggested and I can see that he’s seeing it from the medical [point of view], but I was healthy. I said, are my ovaries healthy? He said yes. So I don’t want to do that, and that was over a year ago.*
23.	Amy (32, tested 8 years ago)	*When I found out my sister was having the ultrasound I said to her, why are you having that? I’m pretty sure in that initial meeting when we all found out that we were carrying it, it was just the colonoscopy that was the screening. There were no other options at that stage…But I will be looking into that.*
24.	Gaby (51, tested 10 years ago)	*[The risk of endometrial cancer] wasn’t mentioned, I don’t think so. I don’t remember. It might have been but I don’t remember…I was bleeding a lot…I went and saw my Oncologist, he said it’s all related. I thought it was more so the breast and ovarian cancer was linked with the bowel, didn’t know about the endometrial. So yeah that was a little bit of a piss me off because I try to keep up with everything. It is what it is, can’t change it, just deal with it.*

Two (22%) female carriers could not recall ever having a discussion around GC screening and risk management (quotes 23 and 24). Amy had learnt about the associated risk of GC through her sister, also a carrier, and then at a recent LS workshop. Gaby didn’t learn about the associated risk of GC until she was diagnosed with endometrial cancer. Both Amy and Gaby adhered to CRC screening recommendations.

These insights demonstrate the perception of lack of clarity available to some women about the risk of GC for carriers, and the options for managing that risk. Of particular concern were women closely following the recommendations given to them for managing their high CRC risk, while being unaware of their increased GC risk. Gaby expressed it was unfair that she was diagnosed with endometrial cancer, given her perception that she was following all the appropriate risk management recommendations. In addition to Amy and Gaby not being offered any advice about GC, the remaining seven women recalled three different sets of recommendations (Table 5).

## Discussion

This study provides novel qualitative insights into the transition between receiving genetic risk information for LS and enacting risk management procedures, exploring why some non-carriers continue to have unnecessary invasive screening, and why some carriers do not follow screening recommendations.

Our study was able to explore some of the reasoning behind continued screening in the non-carrier group, which other studies have not been able to determine [27, 28, 32]. The five non-carriers (31%) with a personal history of polyps felt reassured by their continued screening practice to monitor their polyps, and almost all of them understood that their genetic status did not increase their risk of developing CRC. Only one of these non-carriers, Ella, expressed uncertainty about the value of the predictive genetic test, considering that a yet-to-be- discovered gene was potentially responsible for the development of her polyps. Aktan-Collan et al. [33] report that non-carriers who doubt the validity of predictive genetic testing for LS are more likely to have continued colonoscopies, although they also speculate that medical reasons could be a reason behind continued colonoscopies. Our qualitative data illustrates that although disbelief in the value of the genetic test was present, the main reason behind continued colonoscopies in this group of non-carriers was indeed medical. In fact, only two (13%) non-carriers were having screening colonoscopies, and both were following a recommendation to do so from their gastroenterologist. Given that we collected information on self-reported screening, it is possible that these two non-carriers were having colonoscopies for medical reasons not mentioned here. That said, knowledge about hereditary cancer syndromes and their associated surveillance procedures has been shown to be insufficient amongst non-genetic health professionals, including gastroenterologists [34–37], and clinician recommendation has been found to be a highly influential factor in the uptake of colonoscopies [38]. It is therefore also possible that these gastroenterologists were not knowledgeable enough about LS to understand that ongoing screening colonoscopies are not recommended for asymptomatic non-carriers, even if they have a strong family history of CRC.

As demonstrated by Meagan and Michael, receiving a negative mutation result can take time to adjust to, especially for individuals who have been living for many years as though they were at high risk. Their comments provide qualitative insight into non-carrier cancer-specific distress, which has been shown to steadily decrease in the years following predictive genetic testing for LS [27, 33, 39, 40]. Our findings exemplify that although cancer-specific distress and anxiety generally decreases in the years following predictive genetic testing, non-carriers can experience long-term uncertainty regarding CRC risk management.

A number of non-carriers could not recall what they had been advised regarding population-based CRC screening, and although these non-carriers understood that they were now considered to be at population risk, they were unclear about what population CRC screening entailed and at what age it commenced. This could be due to recall bias, as the only non-carrier who was able to recall this information was Michael. However, given that there was poor knowledge and that genetic result disclosure sessions were reportedly brief in many instances, it is also worth considering investing more time in supporting the psychological and behavioural adaptation of individuals who receive a negative result following predictive genetic testing for LS.

All carriers received a clinical recommendation to have a colonoscopy every 1-2 years. The minority who received conflicting recommendations were not confused or deterred from having regular screening. Our data suggests that in order to adhere to screening recommendations, carriers need to believe they are at increased risk of developing CRC, be able to tolerate the procedure, and believe in the efficacy of colonoscopy screening. One of the two carriers screening sporadically lacked all three of these conditions (Fred), and the other (Matt) lacked two. It is not clear what led these men to have reduced faith in colonoscopy for the prevention of CRC, however this finding is consistent with previous studies on adherence to regular colonoscopies, which note that the number of physical and psychological barriers to screening is related to more sporadic screening [41–44]. The qualitative perspective provided by our study also suggests that some barriers may be more influential than others. For example, Fred declared that even if his screening was organised on his behalf it probably wouldn’t change his screening practice. Rather, it was his poor relationship with his doctor, his disbelief in being at increased risk, and his view that colonoscopies would not reduce his risk of developing CRC which influenced his non-adherence to screening recommendations. Likewise, it is possible that Matt’s lack of faith in the procedure’s ability to reduce his risk has more influence on his screening practice than his dislike for the procedure.

The majority of female carriers (78%) reported some form of GC risk management, either by the way of surgery (prophylactic hysterectomy and BSO) or annual screening (pelvic ultrasound). Of concern, two (22%) female carriers could not recall receiving GC risk management advice (Gaby and Amy). Both of these female carriers were having annual colonoscopies, highlighting a lack of communication about their GC risk. Additionally, although Shelly reported having annual pelvic ultrasounds, she was of the belief that a prophylactic hysterectomy and BSO wasn’t warranted while her ovaries appeared healthy. Given that to date no benefit has been shown for ovarian cancer screening, and there is little evidence for the benefit of pelvic ultrasounds for endometrial cancer screening, Shelly’s comments raise the question of whether she has been accurately informed that the only effective intervention to reduce her risk of GC is prophylactic surgery. Likewise, although Linda and Ruth were having annual pelvic ultrasounds, they were not aware of prophylactic hysterectomy and BSO as a risk management option.

The limited number of studies looking at why adherence to GC risk management is generally low have reported that female carriers are less aware about their associated risk of GC, and therefore less likely to adopt appropriate risk management behaviours [22, 42, 45]. The only reason for not adhering to GC risk management recommendations in our study sample was lack of awareness. Given the high risk of developing GC and the lack of effective screening, it is essential that female carriers are made aware of their increased risk of GC and are fully informed about their risk management options.

## Conclusion

Findings from this study highlight that although the majority of carriers and non-carriers of LS tend to adapt to appropriate CRC and GC risk management in the long term, there is evidence of a hierarchy of confidence in information provided to those receiving predictive genetic test results. Participants expressed most confidence about the use of regular colonoscopies to manage CRC risk for carriers, and less confidence about the risk and management of GC for female carriers, with very few non-carriers able to recall advice regarding population screening. Despite the higher levels of confidence in colonoscopy for carriers, we found evidence of non-adherence to colonoscopy screening. Evidence presented here suggests that to address this, further elaboration of the effectiveness of colonoscopies in reducing the risk of CRC and the meaning of predictive genetic test results will be required for some.

While the relatively small sample size limits the generalisability of findings, saturation of the main themes was evident, suggesting the potential for reproducible findings in future studies. A larger sample size and quantitative methodology would allow for individuals to be grouped based on characteristics such as age and time since testing.

### Practice implications

Our findings, around how genetic risk information for LS translates to risk management behaviours, have implications for the communication of information between specialist cancer genetic services, the patient, and the clinicians responsible for their ongoing management.

Non-carriers:

- Invest more time in result disclosure sessions, ensuring that non-carriers are aware of the recommended screening for their risk level
- In the letter to managing clinicians, explain that a strong family history is not of significance to individuals with a negative mutation result, and that ongoing screening colonoscopies are no longer required for those who are asymptomatic

Carriers:

- Offer additional genetic counselling to carriers not screening regularly to explore the reasoning behind their non-adherence to screening recommendations
- Ensure that female carriers and their managing clinicians are aware of the associated risk of GC and appropriate risk management options
- Communicate with carriers and their managing clinicians when risk management recommendations are updated
